# The Perceived Impact of the Namaste Care Family Program on Nursing Home Residents with Dementia, Staff, and Family Caregivers: A Qualitative Study

**DOI:** 10.1177/08258597221129739

**Published:** 2022-10-05

**Authors:** Hanneke J.A. Smaling, Anneke L. Francke, Wilco P. Achterberg, Karlijn J. Joling, Jenny T. van der Steen

**Affiliations:** 1 Department of Public Health and Primary Care, 4501 Leiden University Medical Center, Leiden, the Netherlands; 2 University Network for the Care sector Zuid-Holland, 4501 Leiden University Medical Center, Leiden, the Netherlands; 3 Department of Public and Occupational Health, Amsterdam University Medical Centers, Location VU University Medical Center, Amsterdam, the Netherlands; 4 8123 Netherlands Institute for Health Services Research (NIVEL), Utrecht, the Netherlands; 5 Department of General Practice and Elderly Care Medicine, Amsterdam University Medical Centers, Location VU University Medical Center, Amsterdam, the Netherlands; 6 Department of Primary and Community Care, Radboud University Medical Center, Nijmegen, the Netherlands

**Keywords:** Namaste Care, dementia, nursing home, person-centered care, challenging behavior

## Abstract

**Objective(s):** To examine the perspectives of staff, and family caregivers (i.e., family, friends, and volunteers) on the impact of the Namaste Care Family program on all involved. The Namaste Care Family program is a structured program for people with advanced dementia based on a palliative care approach in which family caregivers provide daily sessions together with staff with the aim to increase residents’ quality of life. **Methods:** In this descriptive qualitative study, we interviewed 12 family caregivers, and 31 staff members from 10 nursing homes. Data was analyzed thematically. **Results:** A perceived impact of the program was identified for the residents, staff, and family caregivers. For residents, this included well-being, more engagement, enhanced interactions, changes in energy level, and weight gain. The impact on family caregivers included a more positive view of people with dementia, changes in family visits, mixed feelings during sessions, and mixed changes in relations with all involved. For staff, this included diverse work experiences, shift to more person-centered care (more time and attention for residents, and more awareness), and developing relationships with residents and colleagues. **Conclusions:** The Namaste Care Family program was valued for its observed benefits and shift towards a person-centered care culture.

## Introduction

Due to progressive deterioration in cognitive abilities, people with dementia become less engaged with their environment, and experience a reduced ability to socialize and participate in daily activities.^1‐4^ This places them at risk for isolation and suboptimal quality of life. A lack of activities contributes to challenging behavior in people with advanced dementia, stemming from unmet needs such as boredom, sensory deprivation, and need for social interaction.^5,6^ Offering tailored, meaningful activities can improve persons with dementia's quality of life.^7^

Family often wants to stay involved in the care for their relative.^8,9^ Family involvement increases the wellbeing of the person with dementia, and family may feel more satisfied with the care provided.^9,10^ Their knowledge about their relative can be useful in providing person-centered care.^11,12^ Involving family caregivers (i.e., family, friends, and volunteers) in meaningful activities suitable for the stage of dementia and tailored to the person's preferences may improve family caregivers’ experiences, in addition to increasing residents’ quality of life. However, suitable psychosocial interventions for people with advanced dementia that actively involve family caregivers are scarce.

Staff who provide daily care for people with advanced dementia have a crucial role in addressing palliative care needs to improve their residents’ quality of life. Providing palliative care to residents with advanced dementia poses challenges for staff, like developing specialist knowledge and skills, knowing and understanding the resident, and interdisciplinary collaboration (including with family). When proving palliative care to their residents, staff can feel insecure, inadequate and experience stress, and may lack specialist knowledge and skills.^13,14^ An intervention that may address these challenges^15^ is Namaste Care.

Namaste Care^16^ is a psychosocial intervention for people with advanced dementia based on a person-centered and palliative care approach. This structured program combines sensory stimulation, social interaction, and tailored activities to meaningfully connect with the person with dementia to increase quality of life. Studies on the effects of Namaste Care have been promising. Namaste Care reduced challenging behavior,^17‐20^ anti-psychotics, and sleep medication.^21^ Costs of the program are low.^22‐24^ In the UK, staff welcomed Namaste Care.^20,22,25^ Less is known about the perceived impact on all involved when family caregivers are delivering the program together with staff. This study examined whether staff, and family caregivers perceived an impact of the Namaste Care Family program (NCF) on all involved.

## Methods

### Study Design

This interview study is part of the Namaste study,^26^ in which ten nursing homes implemented NCF, while nine nursing homes continued to provide care as usual. Semi-structured interviews were conducted in the context of a process evaluation along with a cluster-RCT. A secondary analysis was performed on the interviews. The evaluation of the family caregiver involvement is described elsewhere.^27^ The interviews took place between December 2017 and October 2018.

We employed a generic qualitative design with an inductive and descriptive approach,^28^ which investigates people's subjective opinions, attitudes, beliefs, and reflections on their experiences. Generic qualitative design is based on main principles in qualitative research, using methods such as the constant comparative method adopted form established qualitative approaches.^28^ The COREQ guideline was used in reporting about this study.^29^

### Namaste Care Family Program

Staff were asked to identify eligible residents for NCF. Eligible residents had advanced dementia and were unable to participate in regular activity programs, or had moderate dementia with challenging behavior, or had a family caregiver that may benefit from the program.

Namaste Care is a multi-component program that can be applied flexibly to tailor to residents’ individual needs and preferences.^16^ The principles that guide the program are a special environment and a loving touch.^30^ Ideally, 2-h-sessions are offered twice a day in a home-like room with soft music, pleasant scent, and without external distractions. Each session starts with a personalized welcome of the residents. Foods and drinks are offered frequently to increase hydration and caloric intake. Before personalized activities are offered, the residents are checked for signs of pain or discomfort. An expressive unhurried touch approach is used in all interactions between the Namaste provider and the residents. Namaste activities stimulate all six senses, aim to increase comfort and pleasure, and are designed to establish meaningful connections to promote wellbeing. The sessions end with staff personally thanking each participant for their attendance.

The sessions are led by staff. The staff received a 2-h training where they learnt about the principles, purpose, and benefits of NCF, and how to provide a Namaste session. Each nursing home received a toolkit^31^ with a manual and practical tools to help them implement, evaluate, and sustain the program in their nursing home. In the original program, family involvement remains limited to regular family meetings to discuss care.^16^ Active participation of family caregivers in delivering the program is emphasized in our adapted version of Namaste Care.^26,32^ Family caregivers received a leaflet about the program and were invited to an information meeting in which the principles and aims of NCF were explained, practical examples of Namaste activities were given, and they were encouraged to participate in the program. When present during sessions, staff invited family caregivers to join in the activities by explaining how and what they could do (e.g., how to give a hand massage).

### Participants

Family caregivers who participated at least twice in Namaste sessions, and staff who frequently took part in NCF could participate in the interview. No inclusion criteria were formulated for the managers. We aimed to interview at least one manager, staff member, and family caregiver per nursing home. They were invited to participate in the interview via email.

### Data Collection

The interviews took place on a location chosen by the interviewees. The interviews were conducted by three female psychologists with knowledge of dementia care and experience with qualitative research (HS, SD, and a research assistant) 12 months after the implementation of NCF. Three interviews were held with two interviewees together at their request. In two nursing homes, interviews were conducted after 3 months (n = 3; nursing home D) and 6 months (n = 3; nursing home I). These nursing homes prematurely stopped NCF due to problems in the organization.

The interview guide comprised a series of questions based on specific topic areas of interest for the process evaluation (e.g., implementation and evaluation of the program, impact on self and others, motivation to join, family participation, and feasibility of the program in the community). In this study, we focused on the questions that tap into the possible impact of the program on all involved (see Appendix 1), while the transcripts were also screened for possible relevant textual units related to the research question. The interviews were audio-recorded and transcribed verbatim.

### Data Analysis

Using an inductive approach, a thematic analysis was performed. Two researchers (HS, SD) each independently read and re-read the transcripts to select relevant textual units (i.e., passages that included data regarding the perceived impact of NCF on all involved). Textual units then were coded, and further abstracted to generate themes.^33^ Based on the first three interviews, codes and themes were identified. The researchers compared and contrasted codes and themes, resulting in an initial coding framework. This was used to recode the first three interviews and to analyze the next three transcripts. When new codes or themes emerged, they were added to the coding framework. The researchers engaged in an iterative process of discussing and refining codes and themes to enhance analytical rigor until all interviews were analyzed. Coding was supported by the software program ATLAS.ti, version 7.5.18 (Scientific Software Development GmbH, Berlin, 2017).

### Ethical Considerations

Written informed consent was obtained from all interviewees. The study protocol has been reviewed by the Medical Ethics Review Committee of the VU University Medical. The study was regarded as a service evaluation and thus deemed exempt from the Medical Research Involving Human Subjects Act (protocol no. 2016.399). The study is registered in the Netherlands Trial Register (NTR5692).

## Findings

Of the 56 people invited, 44 people were interviewed for the process evaluation. One interview was lost due to failure of the recording device, resulting in a sample of 10 family members, 31 staff members, and 2 volunteers. Table 1 presents the sample descriptives.

**Table 1.** Sample Descriptives (N = 43).

	M (SD) / n	Range
Age in years	51.6 (12.8)	22 - 84
Female	40	
Respondent type		
Volunteer	2	
Family member	10	
Staff	32	
Family's relation to person with dementia		
Daughter or son	7	
Daughter/son-in-law	1	
Spouse	2	
Profession of staff		
Manager	9	
Nurse	11	
Activity coordinator	7	
Namaste-coordinator	4	
Duration of interview in minutes	51 (15)	27 - 102

*Note*: Namaste-coordinator is the person responsible for the coordination of the Namaste Care Family program within the nursing home.

The interviewees reported an impact of NCF on residents, staff, and family caregivers. Per group, several themes were developed. For residents, these included well-being, more engagement, improved interactions, changes in energy level, and weight gain. The impact of NCF on residents was mainly observed during the sessions. The impact on family caregivers included a more positive view of people with dementia, changes in family visits, mixed feelings during sessions, and mixed changes in relations with all involved. For staff, the impact included diverse work experiences, shift to more person-centered care (with subthemes more time and attention for residents, and more awareness), and developing relationships with residents and colleagues. Data saturation was reached as no new codes were identified in the final interviews.

### Perceived Impact on Residents

#### Improved Wellbeing

According to the family caregivers and staff, the personal attention during the sessions improved residents' mood. Residents were enjoying the activities. They seemed happy, open, and better able to express emotions than before NCF. The calm, distraction-free environment brought more peace and relaxation among the residents. Bodily tension was reduced, especially when massages were offered. Challenging behavior decreased.

*Most of our residents have stopped the oxazepam, medication to relieve agitation. The ward is much calmer, because you offer activities*. (Staff, nursing home [NH] H)

Overall, the perceived impact on wellbeing was positive, however, two situations were mentioned that could lead to adverse reactions of residents. Incidentally residents became agitated during NCF, which staff ascribed to offering the resident the ‘wrong’ kind of activity:

*We have singing with the minister, which is singing psalms. Many residents liked that. But there was one gentleman who reacted very emotionally and would become very agitated. […], because apparently it did not have pleasant associations for him*. (Namaste coordinator, NH D)

In nursing homes that conducted the sessions in a special Namaste room, some residents were struggling with the transition back to the ward, resulting in agitation. This was attributed to the difference in energy, pace, and personal attention.

#### More Engagement

Residents were more actively engaged in activities during NCF than during ‘regular’ activities, for example when singing, listing to live music, creating music together, coloring, and reminiscing. Numerous anecdotes were told about unresponsive residents who started to participate in activities during NCF:

*The lady just lay in bed in her room. We took her to the sessions and everyone was like, is this a person who should participate? Is there any value to this? And then when the music was playing, we saw that she started to tap along a little with her index finger and just got a very content look on her face*. (Namaste-coordinator, NH D)

A few family caregivers and staff noticed that residents showed more initiative by asked when they could do certain activities. As a spin off, staff also offered residents more activities outside NCF to keep them engaged.

#### Enhanced Interaction

In general, the ambiance on the ward improved. Residents started to laugh, sing and talk more. They were kinder and more lenient towards other residents, resulting in less arguing. More solidarity developed and the ward started to feel more ‘homelike’. While some did not observe any difference in the frequency of interactions between residents, others indicated that the residents interacted more with each other.

*At first everyone was snoring in their chair and that was it. My mother always said ‘this is so boring’. But the atmosphere is just different. You can feel that, and the people are also a little different there. Well, maybe a little more friendly towards each other, a little more tolerance for each other*. (Family caregiver, NH B)

Staff and family caregivers mentioned that NCF enabled them to better connect with residents, also those in the very advanced stages of dementia. Staff and some family members felt that real-life stuffed animals and dolls were useful tools to establish meaningful connections. Residents also expressed their appreciation for Namaste activities.

#### Changes in Energy Level

In the startup phase, residents were usually exhausted after the sessions. They needed time to get used to the additional attention and activities. This adjustment period generally took about a month. After that, it seemed that the impact of the program on energy levels depended on the type of activities offered.

*He walked a lot, wandered a lot. During the Namaste sessions he was present, content and quiet. But as soon as the session was over, the restlessness returned. At that moment it seemed as if he hadn't burned off enough energy during the day, because he would continue during the night*. (Namaste-coordinator, NH D)

#### Weight Gain

In a few nursing homes, residents started to eat better thereby gaining weight. In one nursing home, staff made snacks the focus of the sessions, resulting in too much weight gain. To address this, the amount of snacks was reduced and more healthy alternatives were chosen. The focus also shifted from snacks to activities with the residents.

### Perceived Impact on Family Caregivers

#### Changes in Family Visits

For half of the family members, NCF had no effect on how they experienced the visits to their relative or the frequency of their visits, especially for those who already visited their relative daily. Others visited more often, on a more structural basis or rescheduled their visits outside NCF to ensure their relative received even more personal attention. Some family members felt more positive about the visits to their relative since the introduction of NCF due to the more homelike ambiance on the ward. Family felt encouraged to do more and try out other activities with their relative.

#### Mixed Feelings During Sessions

In general, family caregivers enjoyed participating in NCF. They loved the meaningful contact with the residents and being able to do something together. Family caregivers appreciated the moments when the residents were enjoying themselves or responded positively to the activities. Some family members mentioned that NCF gave them more peace of mind:

*Because Namaste obviously had a positive effect on my mother, it also makes it a little easier for me to let her go. If I saw that she was enjoying herself, then it was OK. And in the beginning I often went to see her, to check ‘oh, how is she?’, but in the end I didn't really do that so much*. (Family caregiver, NH B)

A few negative experiences were mentioned. Family caregivers felt insecure in the startup phase; it was not clear to them whether they were welcome to join, what they could do during NCF, and how to deal with residents wanting to leave the session. With the help of staff and gaining more experience over time, the feelings of insecurity decreased. Participating in the sessions was also confronting for a few family members because they saw residents in more advanced dementia stages or because the response of their relative was painful for them:

*I walked up with the doll and he saw that and I saw his face relax and become open. He just stroked the doll's cheek with his finger. [….] His daughter came in and that was kind of a moment,. To her it was just very shocking, because he never did this with them or the grandchildren. So it's kind of a blow to see your father with a doll and on top of that showing so much affection towards the doll*. (Staff, NH B)

#### More Positive Views of People with Dementia

Family caregivers’ views of people with dementia became more positive through NCF as it increased their awareness that people with dementia are still able to learn new things, and that they can do more than they thought. For some, it was a revelation that people with advanced dementia were still able to enjoy themselves.

*When you’re sitting there and you see this little old lady just talking about the old days. And she realizes….* *’Hey, I can do this. I can still talk about this. I just know it.’ Other people are listening. Then I think, you still have so much value. Even if you don't remember much anymore. In my opinion, people with dementia are also valuable people. There are still some things they can do*. (Family caregiver, NH D)

#### Mixed Changes in Relations with all Involved

Some family caregivers experienced no change in their relation with staff, while others experienced more personal contact with staff because they were seeing them more often. Most family experienced no difference in the quality of the relationship with their relative. Family caregivers felt they got to know the other residents better than before NCF. Specifically, volunteers felt a stronger bond with the residents and enjoyed the contact with other volunteers:

*I thought it was very special that your relationship with residents becomes stronger. Yes, I noticed that [resident] actually didn't have much contact with me. And that has gradually grown a lot, like she would immediately come to me when I entered the room*. (Volunteer, NH G)

### Perceived Impact on Nursing Staff

#### Diverse Work Experiences

Overall, staff indicated that NCF improved their work experience. They appreciated the program because it added something to their work, they were proud of what they accomplished, and enjoyed the quality time with the residents. Residents’ positive responses were gratifying and made them go home feeling good. Most staff experienced the program as relaxing, away from the normal hectic pace on the ward.

*Namaste is fantastic to do. It's just really beautiful to see, it's really a moment of relaxation for me, you are obliged to just sit quietly. And the music makes it peaceful. You take it easy*. (Manager, NH E)

Managers who did not actively participate in NCF reported no impact on their work experience.

In two situations, NCF was considered stressful by some staff, namely during the startup phase and when being understaffed. The stress in the startup was mainly because staff felt insecure:

*At first, it was really finding your way and especially the first month it was also quite exhausting. You had all these questions like ‘am I doing it right?’ and ‘how do the others do that?’. It can make you quite insecure sometimes. And then you feel all eyes are on you and you are the activity coordinator, so you know how we are supposed to do this*. (Staff, NH C)

#### Shift to More Person-Centered Care

NCF made staff more aware of what they were doing and made them critically review their way of working. It was an eye opener for staff that residents with dementia could do more than they initially thought.

Staff felt that the focus of their work shifted from being task-orientated to person-centered. NCF provided them with a legitimate reason to spend more time with the residents giving them one-on-one attention, during and outside NCF, apart from performing their routine care tasks. This was something staff really appreciated and it increased the knowledge about their residents. It also created more awareness for what individual residents need and want. In time, the person-centered NCF activities were incorporated in daily care routine.

#### Developing Relationships with Residents and Colleagues

Staff indicated that team spirit increased by sharing more fun moments and positive interactions in NCF. They experienced improved contact with colleagues and with residents, and developed a closer bond with some residents. Interestingly, family did not see changes in the relation between staff and their relative.

## Discussion

This study found that NCF mostly had a perceived positive impact on all involved during the sessions, according to staff and family caregivers. On some aspects not all interviewees experienced a change and a few negative experiences were mentioned. These negative events were related to circumstances that are preventable or can be reduced. The qualitative data presented here suggests that NCF has much to offer.

The perceived impact on residents included their well-being, more engagement, improved interaction, changes in energy level, and weight gain. Previous studies with the original Namaste Care have also shown a positive effect of the program on challenging behavior,^17‐21,34^ mood,^25,35,36^ engagement,^22,25,34,36^ interaction,^25,34^ and quality of life.^25,37,38^ The calm environment along with the undivided personal attention and meaningful activities may have contributed to this, as others have suggested.^39,40^ Incidentally, NCF had a negative impact on residents, which was mostly due to them needing to get used to NCF or could be ascribed to a mismatch between the activity offered and the resident's preferences and needs. Table 2 describes practical recommendations for providing NCF, based on the our findings.

**Table 2.** Practical Recommendations for the Namaste Care Family Program (NCF) Based on the Qualitative Findings.

Challenge	Recommendation
All involved needed to get used to NCF.	• Allowing a period of supervised trying may facilitate the implementation^27^.
	• Give it time: the full effects of NCF may be more apparent after the startup period.
Residents are struggling with the transition from the dedicated Namaste room back to the ward due to the difference in pace and environment.	• Have a volunteer or staff member welcoming residents back on the ward and spend some time with them to ease the transition.
Staff experience stress when having to provide NCF while being understaffed.	• Increase family caregiver involvement in the sessions.
	• Involve the volunteer coordinator – if present in the nursing home - in planning the Namaste sessions.
Choosing activities for residents that best match their interests, needs, and abilities can be challenging.	• Know the residents (e.g., use life story books, short synopsis per resident with their [dis]likes, [previous] interests, and needs that is continuously updated).
	• Pay attention to possible non-verbal cues^14^.
	• Take needs for stimulation versus relaxation into account to avoid possible adverse effects.
	• Help family and volunteers to recognize which activities could be meaningful for residents, for example by educating them and leading by example.

*Note*: numbers in superscript refer to the references used.

This is the first study showing that NCF, the adapted program, has a perceived impact on all involved. An interesting discrepancy in perspectives was that family did not see changes in the relationship between staff and their relative that staff reported. Perhaps during the sessions, family focused on their interaction with their relative overlooking more subtle and subjective experienced changes in staff-resident relationships becoming stronger and more personal. It could also be that the improved staff-resident relationships were more apparent outside of the sessions, making it less visible for family. Alternative explanations may refer to triadic relationships with competing roles^41^; one-to-one attention of staff may leave the third party (family) feeling excluded.^42^ Some family members may experience a degree of envy. More research is needed to substantiate these possible explanations.

Connecting with people with dementia has been associated with changes in attitudes,^43,44^ and a lower level of stigma.^45,46^ However, there are also indications that more contact may not always be enough to change images.^47^ The quality, duration, and nature of the contact may play an important role in changing stereotypes and reduce stigma. It could be that NCF reduced the sense of ‘otherness’ with regard to people with dementia through its focus on meaningful connections, enjoyable moments, and on the capabilities of people with dementia rather than their impairments. This suggests that staff and family caregiver involvement in a daily care program, and positive, meaningful interactions may help alleviate dementia-related stigma.^48,49^ Future studies should look into the conditions under which increased contact leads to stigma reduction and the potential mechanisms at play.

Most family caregivers experienced changes in relationships with all involved. There were a few family members who did not experience an impact of NCF on their own mood or relation with staff or their relative, despite positive experiences during the sessions. These family members were already frequently involved in the care for their relative before NCF or only participated a few times, thereby experiencing limited additional value of NCF on these aspects. The impact of the sessions on family members’ feelings differed between individuals. It depended on the residents’ responses, and it may depend upon family dynamics, and the grieving process of the family member. It may also relate to a sense of insecurity about the ‘unknown’ of participating in a new program. Coaching from staff and more experience over time may help overcome this.^27,36^

For staff, NCF generally had a positive impact on their work experience. This is in line with previous research.^22,25^ The themes identified for staff are connected and capture a shift to person-centered care through increasing their awareness of the importance of residents’ wellbeing and personal attention, knowing their residents better, and establishing or improving bonds with family caregivers.^34,38^ This indicates that NCF may be a suitable intervention to address some of the challenges nursing staff experience when providing palliative care to nursing home residents with advanced dementia.^13‐15^

A strength of our study includes using various perspectives by interviewing different stakeholders from all nursing homes, also the ones that prematurely ended the program. Limitations include the female staff sample, low number of volunteers, and having to rely on the manager selecting staff and volunteers who met our inclusion criteria. Despite the researchers stressing the importance of also asking candidates who were critical about NCF, we cannot rule out that managers were inclined to ask people who would provide a positive account of NCF. The nature and limitations of the study restrict making definitive assertions regarding the efficacy of NCF. However, this study adds to a growing body of qualitative evidence indicating a predominantly positive impact of the program on all involved^22,25^ and highlights the need for more large-scale RCTs.

## Conclusions

This study identified the importance of ensuring social care is intrinsic in facilities caring for those with advanced dementia, as opposed to just sites of physical and medical care. The structured approach of NCF supports staff to meet the needs of residents with advanced dementia using a person-centered approach and aids family caregivers to meaningfully connect with the residents. Elements of NCF were carried over to every day care. The perceived impact of NCF on all involved was predominately positive. The few negative effects were mostly seen in the startup phase and when offering less suitable activities. These could be dealt with by adjusting the offered activities and easing the transition from Namaste back to the ward. Family caregivers and staff believed benefits will be a catalyst for further implementation of NCF and serve as advocacy for good dementia care.

## Abbreviations

NCF: Namaste Care Family program
NH: nursing home
RCT: randomized control trial.

## Appendix 1 Interview questions

### The Perceived Impact of the Namaste Care Family Program on Nursing Home Residents with Dementia, Staff, and Family Caregivers: A Qualitative Study

- ***General questions for all interviewees***
  1. What was it like for you to participate in the Namaste sessions?*Probes*: What effect did it have on you and why? To what extent does the Namaste Care Family program fit in with your own values, interests, beliefs and preferences?
  2. What do you think it is like for the resident(s) to participate in a Namaste session? Why do you think that?
  3. Can you describe a situation with regard to Namaste that you remember most clearly (positively or negatively)? What specifically made an impression on you?
  4. Have you seen effects of the Namaste Care Family program?*Probes*: Ask about perceived effects on residents, staff, family, effects outside the sessions, relationships (between residents, staff-resident, family-resident, within family).Ask about positive and negative effects.Ask about impact on behavior, mood, health, medication, burden.
  5. Could you describe a moment that you think best reflects the effect of participating in the program for the person with dementia?*Probes*: Why do you choose this moment? What effect did you see?
  6. Which parts of the Namaste Care Family program do you find most valuable and why? *Probe*: What do you feel has the greatest impact?

- ***Only for family members***
  1. Has the Namaste Care Family program influenced the contact between you and the staff? *Probes*: If so, please elaborate. Have you also noticed an effect on the contact between staff and your relative?
- ***Only for staff***
  1. Does the Namaste Care Family program influence how you experience your work?*Probes*: Do you experience your work (or parts of it) differently than before the implementation? If so, what things exactly and why is that?

The full interview guide can be found here DOI:10.1016/j.ijnurstu.2021.103968.
